# Politics, diplomacy, and the Asian games

**DOI:** 10.3389/fspor.2026.1882064

**Published:** 2026-07-15

**Authors:** Yicai Yu, Fan Hong

**Affiliations:** Institute of Olympic Studies and Research, Shanghai University of Sport, Shanghai, China

**Keywords:** Asian games, sports diplomacy, host politics, national self-representation, recognition

## Abstract

**Introduction:**

The Asian Games are often discussed as a regional counterpart to the Olympics, yet their political significance remains insufficiently examined. This article addresses this gap by asking how host states use the Asian Games as a political platform.

**Methods:**

The study draws on a comparative analysis of recent host cases, including China, South Korea, Indonesia, Qatar, and Japan. It uses official documents, government promotional materials, media coverage, and firsthand observations to examine the political meanings attached to hosting.

**Results:**

The article argues that host states use the Asian Games to advance political claims through the act of hosting itself. More specifically, they use the event to shape national self-representation, stage diplomatic signaling, and seek external recognition.

**Discussion:**

The article shows that the political significance of the Asian Games lies not simply in the images and messages that hosts seek to project, but also in the responses these efforts generate. By shifting attention from sport as display to sport as a process of political negotiation, the article demonstrates that the Asian Games offer a distinctive lens through which to understand how identity, diplomacy, and recognition are contested in contemporary Asia.

## 1 Introduction

Sport has long been intertwined with political aspiration. From the Olympic Truce in ancient Olympia to the modern rhetoric of peace, unity, and friendship surrounding global sporting events, sport has repeatedly been used for political ends (1). Because major sporting events attract wide public attention and strong emotional investment, they give states highly visible platforms from which to project power, shape national identity, and pursue diplomatic goals (2). Scholarship has examined the relationship between sport and politics extensively, especially through the Olympic Games. Research on sports diplomacy, soft power, and nation branding has produced a substantial literature on global sporting mega-events (1, 3–9, 51, 60, 61). By contrast, the Asian Games have received far less scholarly attention, despite being the world's second-largest multi-sport event. This neglect is striking given the scale of the competition: the Hangzhou 2022 Asian Games, held in 2023 because of the COVID-19 pandemic, brought together over 12,000 athletes across 40 sports and 481 medal events, while Paris 2024 featured 10,813 athletes across 32 sports and 329 medal events (63, 64). The relative lack of attention matters because the Asian Games are not simply a regional version of the Olympics. Unlike the Olympic Games, which are often framed through a universal global narrative, the Asian Games are more deeply embedded in the politics of regional identity formation, postwar state-building, and shifting power relations in Asia. Their institutional origins can be traced back to the Far Eastern Championship Games held between 1913 and 1934, which already reflected changing hierarchies and intensifying tensions in prewar Asia, particularly in the context of Japanese imperial expansion (10). From the outset, then, regional sporting events in Asia were never politically neutral.

The first Asian Games, held in New Delhi in 1951 during the early Cold War, reinforced this pattern. However, these Games were more than just a sporting event. Coming only four years after India's independence from Britain, they represented a collective statement from newly independent Asian states, such as Indonesia, The Philippines, India, Pakistan, etc. seeking to articulate a shared identity and a vision of peaceful coexistence amid decolonization and ideological confrontation. Yet Asia's regional context differed fundamentally from the path of integration pursued in Europe. Rather than institutional convergence and political homogeneity, Asia has been characterized by extreme diversity. The region encompasses a wide range of political systems, levels of economic development, and social structures (43). It is marked by unresolved historical grievances, territorial disputes, ideological competition, and persistent strategic mistrust (62). At the same time, Asia has witnessed some of the most dramatic shifts in global economic power in recent decades (11). In such a context, the political significance of the Asian Games lies not in any presumed separation between sport and politics, but in the fact that the Games make their entanglement especially visible.

This article starts from that premise. In Asia, sport, diplomacy, and politics are not accidentally connected; they are deeply intertwined, and the Asian Games intensify this entanglement in particularly visible ways. This raises the central question of the article: how do host states use the Games to project national identity, conduct diplomatic signaling, and seek recognition from regional and wider international order?

Methodologically, this study draws on a combination of primary and secondary sources to examine the political and diplomatic dimensions of the Asian Games. Primary materials include official documents, government-released promotional texts, and media coverage from recent editions of the Games. The selected cases, including China, South Korea, Indonesia, Qatar, and Japan, are chosen to focus on recent examples that reflect diverse geopolitical settings, host-state types, and political strategies in contemporary Asia. The study also incorporates first-hand observations accumulated by one of the authors, who served as an administration and management staff in one of the most recent Asian Games (Hangzhou) Organizing Committee for two years. During this period, she maintained regular contact with both the preceding Jakarta 2018 Asian Games Organizing Committee and the subsequent Aichi-Nagoya 2026 Committee, gaining insight into internal planning processes, cross-organizational coordination, and the strategic messaging surrounding the Games. Secondary sources include academic studies mentioned in the references, historical analyses, and relevant policy reports. These materials are examined through comparative and textual analysis, with particular attention to host-state narratives, diplomatic practices, and the interaction between sport and regional politics in Asia.

## 2 From sport to politics: rethinking the Asian games as a political arena

The relationship between sport and politics has long been recognized in academic scholarship, but earlier studies tended to treat that relationship primarily through the lens of the nation-state and ideological confrontation. In the context of the Cold War and earlier forms of geopolitical rivalry, sport was often understood as an extension of political systems and state power. Hoberman (60) showed that modern sport became deeply embedded in the nation-state system, serving not only as a source of collective identity but also as a medium through which political values and ideological superiority could be performed. Houlihan (7) further demonstrated that sports policy is often incorporated into broader state strategies, including social cohesion, international prestige, and diplomatic positioning. Likewise, Guttmann (6) emphasized that the modern Olympic Games have never been merely about athletic competition; they have also functioned as symbolic sites for nationalism, international recognition, and struggles about power. Allison (61) extended this line of argument by stressing that sport is international not only because competitions cross borders, but also because international sporting organizations themselves are embedded in wider structures of global politics.

After the Cold War, scholarships on sport and politics expanded beyond ideological rivalry and increasingly engaged with globalization, soft power, and public diplomacy. In this context, sport came to be understood less as a direct extension of coercive politics and more as a form of attraction, image-making, and symbolic influence. Grix (1) argued that mega-events such as the Olympics and the FIFA World Cup became particularly attractive to emerging states because they offered a relatively rapid means of enhancing international visibility, showcasing modernity, and competing for global influence. Murray (42) further refined this discussion by defining sports diplomacy as the conscious, strategic, and ongoing use of sport, athletes, and sporting events by both state and non-state actors to advance policy, trade, development, education, image, and reputation. This shift was important because it moved the literature from viewing sport simply as a reflection of politics to understanding it as a practical instrument through which states and other actors actively operate in international affairs. At the same time, Chappelet's work usefully broadened this debate by showing that the politics of contemporary sport also lies in governance and regulation. His studies on international sport regulation and Olympic governance emphasize that mega-events are shaped not only by image and diplomacy, but also by complex relations among governments, sporting bodies, athletes, courts, and other stakeholders (4, 12, 13). His more recent work on Eastern Asia's integration into the Olympic system further highlights how sport in Asia is embedded in longer-term institutional and geopolitical processes (14).

In the Asian context, Cha (3, 15) was among the earlier scholars to highlight the distinctiveness of the relationship between sport and politics in the region. He argued that, in Asia, where symbolic meaning, political subtlety, and diplomatic signaling carry exceptional weight, sport becomes especially significant in the making and conduct of policy. Rather than treating Asian sport merely as a regional subset of Olympic politics, he showed that it is closely bound to nationalism, diplomatic maneuver, and the pressures of globalization (46). Sporting events in Asia, in his account, are not marginal cultural spectacles but highly charged political arenas in which states project identity, test diplomatic openings, and confront the consequences of political change. Cho and Leary (16), working from a postcolonial perspective, highlighted the close connection between the spread of modern sport in Asia and the intertwined processes of colonization, decolonization, and nation-building. In this view, sport was not only a colonial instrument but also a means through which local actors articulated resistance, modern identity, and national belonging. Hong and Zhouxiang (5) likewise demonstrated, through the case of China's Olympic strategy in the 1980s, that Asian states have repeatedly incorporated sport into broader projects of modernization and international positioning. Fan Hong's edited volume Sport, Nationalism and Orientalism: The Asian Games (10) was especially important because it placed the Asian Games themselves at the center of academic inquiry. Rather than treating them as a secondary or regional copy of the Olympics, this work analyzed the Games as a distinct political formation shaped by nationalism, identity, power struggle, and competing understandings of Asia. In doing so, it established that the Asian Games are not simply sporting occasions held in Asia, but key sites through which Asia is politically imagined, contested, and represented.

More recent studies have further connected the Asian Games to broader transformations in regional and global politics. Black (2) and Aryal (11), for example, emphasize that with the rise of major powers like China and India, the Asian Games have gradually become a symbol of shifting global power. These events are no longer merely platforms for showcasing athletic achievements but have become key arenas for major regional players to compete for influence and power. They have become a significant issue in the ongoing transformation of global politics.

Overall, existing scholarship has examined the Asian Games through broad political themes such as nationalism, imperialism, modernization, political dispute, and state intention, and established the fact that sport cannot be separated from politics, diplomacy, and state strategy. This article builds on that literature by bringing more recent cases into the discussion and by shifting attention to the politics of recognition. Rather than treating the Games only as a reflection of broader political forces, it asks how host states use hosting itself as a political practice and how the meanings attached to that practice are received by external audiences.

## 3 Sport and identity: Asian games as a self-representation platform

Sport and identity are discussed first because identity projection is the most immediate political function of hosting. Before the Games can be used to widen diplomatic space or seek external recognition, they must first provide a stage on which host states present themselves to others. This section therefore examines how selected host states have used the Asian Games as a platform for national self-representation. It focuses mainly on contemporary cases, while retaining several earlier hosting as historical reference points, in order to show how the political uses of the Games have evolved across different contexts. The aim is not to reconstruct the full history of the Asian Games, but to identify how host states use hosting to make political and cultural images of themselves visible.

### 3.1 China: modernity, digital capability, and “Responsible Major Country” identity

China used the Asian Games to project an identity centered on modernity, competence, and the image of a “responsible major country”. Across Beijing 1990, Guangzhou 2010, and Hangzhou 2022, these Asian Games served not simply as sporting occasions, but as stages on which different phases of China's national self-representation were made visible.

The 1990 Beijing Asian Games were especially significant because they were held at a moment of exceptional political sensitivity. China had only recently gone through the 1989 Tiananmen crisis and was facing considerable pressure both at home and abroad. Internationally, relations with many Western countries had deteriorated sharply, sanctions and diplomatic isolation had damaged China's external image, and the end of the Cold War was reshaping the wider ideological environment in ways that heightened uncertainty for socialist states after the fall of the Berlin wall in 1989. Domestically, China was still in the relatively early stage of reform and opening-up, and the leadership faced the dual task of restoring political stability and sustaining confidence in development. As the first large-scale international multi-sport event hosted by the People's Republic of China, the Games therefore carried significance far beyond sport (17, 18). This significance was further intensified by the historical atmosphere of 1990, marked by major anniversaries linked to modern national humiliation, including the 150th anniversary of the First Opium War, the 130th anniversary of the destruction of the Old Summer Palace in 1860, and the 90th anniversary of the 1900 invasion of Beijing by the Eight-Nation Alliance. In this context, the Games offered China an opportunity to project an image of stability, order, and national recovery. They also responded to a strong domestic demand for confidence and cohesion. A successful event allowed the state to present itself as disciplined, capable, and able to guide the country away from crisis and historical weakness toward renewed development. Beijing 1990 thus functioned both as an exercise in international image-building and as a nationalist moment of symbolic restoration.

By 2010, the international environment had changed profoundly. China was no longer operating under the post-1989 pressures of isolation and image repair. After three decades of reform and opening-up, and especially in the aftermath of the 2008 Beijing Olympics and the global financial crisis, China had become far more deeply integrated into the international system and was increasingly perceived as a rising power. The financial crisis weakened confidence in Western economic models and heightened global attention to China's growth, resilience, and state capacity, while China's emergence as the world's second-largest economy further strengthened the sense that the balance of global power was shifting. Official media coverage by outlets such as People's Daily, Xinhua, and CCTV focused not only on the competitions, but also on infrastructure, logistics, construction speed, and urban transformation (53). Narratives such as “China Speed” and the “Chinese Model” framed the Chinese Asian Games as evidence of organizational superiority and national capability (48, 49). In this sense, Guangzhou projected more than national pride. It presented China's political model as effective, coordinated, and able to convert state-led development into visible performance (47). The identity on display was that of a confident rising power whose success rested on competence, scale, and execution.

Whereas Beijing 1990 emphasized recovery and Guangzhou 2010 highlighted efficiency, Hangzhou 2022 highlighted intelligence, digital governance, and future-oriented development. In fact, the Hangzhou Asian Games were not held in 2022 but postponed to 2023. The delay reflected the tension between China's strict zero-COVID policy and wider expectations that such a major regional event should be more open. At the same time, China's closed pandemic controls were increasingly criticized abroad, which made the eventual staging of the Games an opportunity to project renewed confidence and recovery in a distinctly technological form. Hangzhou was not only about staging a successful sporting event; it was also about showcasing a post-pandemic image of “Digital China” as efficient, resilient, and future-oriented. At the same time, China's image as a “responsible major country” was articulated through the official language of green, economical, and sustainable event hosting (50). Official materials framed Hangzhou as striving to build the first carbon-neutral Asian Games, repeatedly invoked the idea of “Zero Carbon,” and emphasized the principle of “reconstructing instead of building, repairing instead of replacing, renting instead of buying.” (19). In this sense, Hangzhou projected a China that sought to appear not only technologically capable, but also normatively responsible in the global language of low-carbon development and sustainable governance. This image was re-emphasized by the World Games in Chengdu, Sichuan, in 2025.

### 3.2 South Korea: diversity, cultural visibility, and middle-power identity

South Korea used the Asian Games (and many other sporting events such as the Olympics, the World University Games, several major world championships) to project an evolving national identity. Across Seoul 1986, Busan 2002, and Incheon 2014, these Asian Games helped recast the country from a developmental state seeking national affirmation into a culturally visible middle power. The language changed over time, but the underlying purpose remained similar: to make the Korean nation appear successful, modern, and increasingly confident on a regional and world stage.

In 1986, South Korea was still operating under authoritarian rule, the Cold War structure on the Korean peninsula remained firmly in place, and inter-Korean rivalry continued to shape the country's external position and internal political logic. At the same time, the country had undergone rapid export-led industrialization and was eager to demonstrate that it had moved beyond the poverty and instability of the postwar decades. Therefore, the 1986 Asian Games mainly served as a “test event” for the 1988 Seoul Olympics and marked the “coming of age of South Korean sports.” (3). That description is revealing. Seoul 1986 did more than showcase athletes. It turned rapid development, organizational discipline, and state-led modernization into a visible public performance. At the domestic level, this mattered because the event helped reinforce regime legitimacy by linking national progress to international success. At the symbolic level, it allowed South Korea to present itself as a country that had moved beyond postwar weakness and could now claim competence, order, and modernity in front of Asia and the world.

By 2002, the international and domestic setting had changed substantially. South Korea was no longer a late-developing state seeking basic recognition. It had democratized, consolidated its position as an advanced industrial economy, recovered from the 1997 Asian financial crisis, and gained greater international confidence through both economic resilience and political transition. Regionally, the early 2000s were also marked by a relatively more hopeful atmosphere on the Korean peninsula, following the 2000 inter-Korean summit and the broader Sunshine Policy period (20). Busan 2002 Asian Games no longer needed to prove that South Korea could host a large event. Instead, it reinforced an image of national maturity. The official slogan, “New Vision, New Asia,” and the symbolic language of the emblem and mascot linked Busan to both Korea and a wider Asian future. The point here was less recovery than self-assurance. South Korea used the Games to show that its earlier developmental success had been consolidated into a stable and forward-looking national image. If Seoul 1986 was about arrival, Busan 2002 was more about confidence. It projected a South Korea that was no longer catching up but was already capable of imagining and representing a broader regional future.

The clearest identity shift came with Incheon 2014. By then, South Korea occupied a different symbolic and geopolitical position. It was already widely recognized as a successful economy and stable democracy, while the rise of the Korean Wave, or Hallyu, had significantly increased its cultural visibility across Asia and beyond.

At the same time, the regional environment had become more complex, shaped by intensifying competition among China, Japan, and the United States, as well as renewed uncertainty on the Korean peninsula. South Korea thus presented itself as open, plural, and regionally connected. The opening ceremony strengthened this identity through a highly visible mix of traditional performance, film direction, and popular culture. Official planning materials for the Incheon opening ceremony emphasized an “all-star cast” and presented celebrity participation to generate early public attention and anticipation for the Games, while outside reporting described the event as K-pop-inspired (52). This gave the Games a distinctly contemporary Korean cultural signature. Sport, in this setting, became a stage not only for athletic competition, but also for national self-display through cultural visibility. What Incheon projected was not simply developmental success, but a South Korea that sought to be seen as culturally attractive, socially open, and regionally legible as a middle power.

### 3.3 Indonesia and Qatar: national branding and aspirational state identity

Indonesia has hosted the Asian Games twice, in 1962 and 2018. Jakarta 1962 was deeply shaped by the postcolonial political environment of the time. Indonesia was still a relatively young state under Sukarno's Guided Democracy, and the Games were used to project national dignity, political relevance, and international legitimacy in the midst of wider political upheaval, economic difficulty, postcolonial nationalism and the non-aligned movement (17, 18, 21). By 2018, Indonesia was operating as a large, democratic, and relatively resilient emerging economy (59), it no longer needed to display sovereignty; it needed to display vitality. Under President Jokowi, the slogan “Energy of Asia” and official calls to show the “friendly face of our nation” framed the Games as a moment of national self-display (58). Ahead of the Games, President Jokowi described them as a major national occasion and urged Indonesians to promote them as a “great national event” (22). At the opening ceremony, he spoke of Indonesia's “pride and honor” in receiving participants from 45 countries (23). Jakarta 2018 therefore branded Indonesia as youthful, confident, and organizationally capable. The point was not simply that Indonesia could host, but that Indonesia should be seen as dynamic and regionally significant.

Qatar used the Asian Games in a similarly aspirational way, but under different geopolitical conditions. Doha 2006 came at a time when Qatar was seeking to transform itself from a small hydrocarbon-rich monarchy on the margins of Asian politics into a more visible and consequential regional actor (24). As the first Asian Games hosted in the Gulf and only the second in West Asia (after Tehran 1974), Doha 2006 gave Qatar an unprecedented continental platform. The opening ceremony functioned as a carefully staged act of national self-introduction, combining Qatari and wider Arab cultural references with technological spectacle to present Qatar as modern, ambitious, and fully engaged with Asia rather than peripheral to it. Doha 2006 was therefore less a one-off event than the opening move in a longer strategy of visibility through sport.

Doha will host the Asian Games again in 2030. After two decades of sustained investment in sport and repeated experience hosting major events, Qatar now presents itself as a state capable not only of organizing the Games, but also of supporting and shaping Asian sport at an institutional level. This is reflected in official Doha 2030 messaging, which highlights “Project Legacy,” extensive existing infrastructure, and early support for Asian National Olympic Committees, rather than framing the event mainly as a moment of symbolic arrival or self-introduction. The election of Sheikh Joaan bin Hamad Al Thani as OCA President in January 2026 further strengthened this institutional projection. Taken together, Doha 2006 and Doha 2030 show a clear shift in identity strategy: from self-introduction to a claim of reliability, influence, and indispensability within Asian sport.

### 3.4 Japan: consensus, openness, and civilian-power identity

For Japan, the central political issue is not capability (of hosting) but legitimacy. In Asia, Japan's material strength remains inseparable from the memory of empire, war, and unresolved historical grievance. For that reason, when Japan hosts the Asian Games, it does not foreground power in a direct way. It emphasizes peace, restraint, openness, and civic order. The function of the Games is to translate these claims into a visible and regionally acceptable national identity.

Hiroshima 1994 was especially significant in this regard. It took place under Prime Minister Tomiichi Murayama, at a time of post-bubble uncertainty, political realignment, and renewed debate over Japan's wartime responsibility. Hiroshima, as the city most closely associated with war memory and nuclear destruction, allowed Japan to foreground peace and reflection rather than national grandeur. This was consistent with the broader Murayama line, which placed unusual emphasis on colonial rule, aggression, and historical responsibility. Hiroshima's own Peace Declarations in 1993 and 1994 explicitly linked the Asian Games to the question of how Japan was viewed by other Asian peoples and to the need not to forget Japan's past domination of Asia (49). In this sense, Hiroshima 1994 was not simply to celebrate peace in the abstract. It was to recast Japan politically: from former imperial aggressor to reflective postwar state; from strategic power to morally restrained regional actor (25).

Aichi-Nagoya 2026 is different, but the identity logic is similar. Official materials define the Games through the slogan “Imagine One Asia” and frame them in terms of connection across language, culture, and nationality (44). The language avoids grandeur and leadership claims, and instead presents Japan as open, orderly, cooperative, and easy to accept. That choice is politically significant. Contemporary Japan no longer speaks only in the idiom of postwar remorse; it also presents itself as a proactive but still civilian-oriented regional actor (26). Precisely for that reason, the Asian Games remain useful. They soften the harder edges of Japan's strategic profile by placing national self-representation in a register of peace, exchange, and coexistence. Hiroshima 1994 did this through memory and remorse; Aichi-Nagoya 2026 does it through openness and regional connectedness. In both cases, the political function is similar: to present Japan not as a threatening power, but as a strong state whose legitimacy rests on restraint, civility, and regional acceptability.

## 4 Sport and diplomacy: the Asian games as a diplomatic instrument

One of the clearest diplomatic functions of the Asian Games is agenda-setting. The event brings together heads of government, ministers, diplomats, sports officials, and delegations in a single regional setting, giving the host an unusual opportunity to arrange meetings, prioritize issues, and shape the tone of interaction. Its value lies not in replacing formal diplomacy, but in widening diplomatic room for maneuver. China used this function clearly in Guangzhou in 2010, when Premier Wen Jiabao met the Pakistani president and the Thai prime minister during the opening of the Games and discussed issues that went well beyond sport, including bilateral ties and regional matters (27). This pattern became even more explicit in Hangzhou in 2023. Before the opening ceremony, China's foreign ministry invited diplomats from more than ten Asian countries to Zhejiang, where they visited cities such as Jiaxing and Huzhou, observed local preparations, and experienced the wider atmosphere of the Games in advance (28). Around the opening ceremony itself, President Xi Jinping used the Hangzhou Asian Games as an occasion for what Chinese official and party-state discourse framed as “home diplomacy” (主场外交), literally diplomacy conducted on domestic ground. The ceremony was formally paired with a welcoming banquet at the Xihu Hotel and several bilateral meetings (54). Among them, Bashar al-Assad's visit was especially significant. His trip to China was his first after the outbreak of the Syrian civil war in 2011. After years of limited foreign travel, the visit marked one of his longest absences from Syria and formed part of a broader effort to break more than a decade of diplomatic isolation under Western sanctions (55). The symbolism therefore extended well beyond sport. By receiving Assad in Hangzhou during the Asian Games, China signaled its willingness to engage a leader still widely shunned in the West, while using the event to demonstrate diplomatic autonomy and to present itself as a power capable of conferring political recognition outside Western-led frameworks. In this sense, the Games served as a concrete diplomatic platform: they gathered Asian leaders in one place, allowed China to shape the pace and format of interaction, and integrated a sporting event into summit diplomacy with effects extending far beyond the Games themselves.

Indonesia also used the 2018 Jakarta Games in this way: its foreign ministry organized an “Asian Games Diplomatic Walk” with diplomats and explicitly presented the event as a platform for expanding friendship (29). Likewise, when Doha secured the 2030 Asian Games, Qatari officials explicitly embedded the event within Qatar National Vision 2030. Official bid materials present the Games not as a stand-alone sporting event, but as part of a broader strategy of international cooperation and sustainable development, through which Qatar seeks to connect sport with economic diversification, regional partnership-building, and a more influential position in Asian sport (30).

A second diplomatic function lies in relationship management through symbolic signaling. The Asian Games can create a low-cost setting in which states test atmospheres, soften posture, and briefly widen room for contact without making formal concessions. The 2014 Incheon Asian Games offer a clear example. North Korea's participation gave the event a significance that exceeded athletic competition. Even before the Games fully began, the arrival of North Korean athletes was staged and received in a visibly symbolic way: supporters at Incheon airport greeted them by waving the Korean Peninsula flag and singing “Bangapseumnida” (Welcome), while some of the athletes waved and smiled back (31). Such scenes did not alter the underlying political structure of inter-Korean relations, but they created a carefully managed public moment in which familiarity briefly displaced hostility. The same logic appeared at the close of the Games. A high-level North Korean delegation, including Hwang Pyong-so, Choe Ryong-hae, and Kim Yang-gon, attended the closing ceremony alongside senior South Korean officials (31). On the same day, the two sides held luncheon talks in Incheon, during which they agreed to continue dialogue and to arrange a second round of high-level inter-Korean talks (Ministry of Unification, Republic of (31). The political importance of Incheon therefore did not lie in any dramatic diplomatic breakthrough. Rather, the Games worked as a symbolic and procedural opening: they provided a legitimate public setting in which gestures of welcome, shared ceremony, and limited official contact could take place. In this sense, the Incheon Asian Games show how sport can function diplomatically not by resolving conflict, but by making limited contact temporarily possible and politically acceptable. The North Korean participation likely facilitated its later involvement in the PyeongChang 2018 Olympic Winter Games, enabling joint marching in the opening ceremony and the formation of a common women's ice hockey team.

The diplomatic use of the Asian Games, however, is not limited to smoothing relations. The event can also become a site of boundary-drawing and political contestation. Sport does not suspend geopolitics; in some cases, it compresses it. The clearest early example remains Jakarta 1962, when Indonesia refused visas to Israel athletes, turning a regional sporting event into a dispute over recognition, legitimacy, and the rules of participation. The interesting fact is that, recently in 2025, Indonesia again denied entry to Israeli athletes, this time at the World Artistic Gymnastics Championships in Jakarta, prompting criticism from the IOC and renewed debate over the limits of political neutrality in sport. A similar dynamic appeared before at the Hangzhou Asian Games in 2023, when the visa dispute involving three athletes from Arunachal Pradesh triggered a diplomatic row between India and China. These episodes are important because they show the limits of sports diplomacy. The Games may facilitate contact and project goodwill, but they can also expose unresolved territorial, ideological, and recognition conflicts with unusual clarity.

What the Asian Games reveal, then, is a distinctive form of regional sports diplomacy. The event works best not as a substitute for foreign policy, but as a supplementary instrument. It can convene actors, transmit signals, and create temporary openings. At the same time, its effects remain conditional. Where political space already exists, the Games may widen it. Where rivalry remains sharp, they may instead reveal its limits more clearly. That is precisely why the Asian Games matter diplomatically: they show not only how sport becomes part of diplomacy in Asia, but also how far diplomacy through sport can actually go.

## 5 The politics of recognition in the Asian games

Host states undertake these above-mentioned efforts to seek recognition. As scholarship in international relations makes clear, political claims do not become meaningful through self-assertion alone. Rather, they acquire force only when they are recognized by others (32, 33). Lindemann and Ringmar (34)) emprise in their book that the pursuit of recognition, and the resentment generated by its denial, is a basic driver of state behavior rather than a marginal feature of diplomacy. Murray (9) further argues that state identities cannot be formed in isolation and that acts of recognition are constitutive of identity because they provide the social authority needed for states to act in ways consistent with their self-understanding. Recognition therefore depends on audience response. In the case of the Asian Games, this means that the political significance of hosting lies not only in what host states seek to project, but also in how regional and wider international audiences receive, accept, qualify, or resist those claims.

Unlike the Olympics, where recognition is sought before a diffuse global public, the Asian Games place the host before a more proximate and politically consequential regional audience, organized through the Olympic Council of Asia and its 45 National/Regional Olympic Committees. That audience is not neutral. It enters the event with historical memory, alliance commitments, strategic rivalry, ideological preferences, and normative expectations already in place. What is at stake, therefore, is not recognition in the abstract, but the uneven validation of competing claims to legitimacy, modernity, responsibility, openness, and regional relevance. The same opening ceremony, diplomatic gesture, or sustainability narrative may be welcomed by friendly audiences, institutionally endorsed by regional bodies, questioned by skeptical observers, or resisted by rivals. Recognition, in this setting, is the social process through which the political meaning of hosting is judged, and the Asian Games provide one of the most visible arenas in which that process unfolds in contemporary Asia.

For example, after the Hangzhou Asian Games, China received strong recognition for organizational competence, technical sophistication, and delivery capacity. Praise for the Athletes' Village, the emphasis on digital innovation, and the presentation of Hangzhou as a “green” Asian Games all reinforced the image China sought to project: that of a capable, modern, and responsible regional actor (35, 36). This recognition was further amplified by friendly and sympathetic audiences. Positive media responses from Pakistan and Laos, together with the attendance of Cambodia's King Norodom Sihamoni at the opening ceremony, helped strengthen the legitimacy of China's hosting moment (28, 37, 38). At this level, the Games did more than display Chinese competence; they generated external acknowledgment of it.

At the same time, some skeptical audiences were far less willing to accept China's preferred political framing at face value. U.S. media commentary, for example, noted that Hangzhou 2022's discourse of unity and harmony unfolded alongside the visa dispute with India, tensions over Taiwan, and confrontation in the South China Sea (39). China's capacity was widely acknowledged, but its broader normative claims were still filtered through pre-existing strategic suspicion and geopolitical rivalry. What was at issue, therefore, was not simply whether China had hosted successfully, but whether that performance could be converted into broader acceptance of China's preferred identity as a responsible and normatively legitimate leading power in Asia. Hangzhou generated recognition for capacity, but only qualified recognition for the broader political image and regional role that China sought to project through the Games.

For Qatar, Doha 2006 was Qatar's first major effort to use the Asian Games to present itself on a continental stage as more than a small hydrocarbon-rich monarchy. With several Asian championships in Doha, the Games gave Qatar visibility, and that visibility mattered politically because it enabled the state to present itself as relevant to Asian affairs rather than peripheral to them. This logic became even clearer in the run-up to Doha 2030, where the emphasis shifted from self-introduction to reliability, infrastructure, and long-term contribution to Asian sport governance. In this respect, the Games offered Qatar a platform through which to seek broader acknowledgment of its relevance and usefulness within Asian sport and regional diplomacy. Yet Qatar's case also shows that visibility invites judgment. The more successfully a state attracts attention through sport, the more intensely it may be scrutinized by audiences it does not control. Recognition, then, is not a linear movement from performance to approval. It is a contingent and contested process in which visibility may strengthen a host's preferred image but may also expose tensions and contradictions that complicate the political value of hosting. The 2022 FIFA World Cup in Qatar is another example of this difficulty (45).

The Korean peninsula case illustrates a different point: recognition is often highly conditional and may be temporary rather than durable. Incheon 2014 showed that the Asian Games can create limited moments of mutual acknowledgment when wider political space already exists. Yet the harder atmosphere surrounding (19) demonstrated how fragile such recognition remains when the broader political environment deteriorates. During the Games, North Korean athletes refused a customary podium group photo with South Korean medalists, and North Korean broadcasts referred to South Korea as “puppets” (40). In this case, what changed was not the formal character of the event, but the surrounding political context (which had deteriorated since 2014–2018). The broader implication is clear: the Asian Games can widen political space when such space already exists, but when confrontation deepens, their capacity to generate recognition is sharply reduced. In this sense, sport does not suspend geopolitics; it exposes the limits within which recognition remains possible.

For the upcoming Aichi-Nagoya Asian Games, Japan's path to external recognition is likely to be more difficult. Japan has long positioned the United States as its closest and most important ally (41), yet the increasingly security-oriented character of that alliance, together with the rise of the domestic right, makes its projected image of peace and friendliness harder to sustain. This difficulty is further intensified by recently deteriorating China-Japan relations, which limit the credibility and regional acceptability of the identity Japan seeks to project. In this sense, the key issue is not whether Japan can host the Games successfully, but whether its preferred self-presentation can still secure wider recognition under current political conditions, despite the “Imagine one Asia” slogan.

To conclude, friendly audiences may reinforce a host's preferred image; institutional audiences may validate competence and delivery; skeptical observers may question the political meanings attached to successful hosting; and strategic rivals may interpret the same performance through pre-existing distrust and unresolved conflict. The Asian Games are therefore not a site of one-way display, where hosts simply present themselves to the world on their own terms. They are a site of political contestation, in which meaning is negotiated between hosts and audiences, and in which the political value of hosting depends on whether projected identities and diplomatic gestures are taken up, qualified, or rejected by others.

## 6 Conclusion

This article tries to answer the central questions (see page 2): how do host states use the Games to project national identity, conduct diplomatic signaling, and seek recognition from regional and wider international order? It argues that host states use the Asian Games to project national identity through carefully crafted slogans, narratives, ceremonies, venues, and technological displays that turn historical memory, political values, and development achievements into vivid national images. They also use the Games for diplomatic signaling by creating a low-threat setting for leader meetings, symbolic interactions, and event-mediated encounters through which they communicate status, strategic preferences, and positions on contested issues. In addition, hosts seek recognition by presenting these identities and diplomatic signals to regional and international audiences. Yet recognition is never automatic, and the political effects of these efforts vary in both force and outcome.

For a rising power such as China, the Games have been used to turn organizational success, technological capacity, and developmental achievement into broader claims to regional authority. Yet the Hangzhou 2022 Games also revealed the limits of that strategy: recognition of capability does not automatically translate into recognition of leadership. For a middle power such as South Korea, the Games have been used less to assert scale than to present the country as a culturally visible and diplomatically flexible regional actor. Incheon 2014 showed that temporary openings for contact cannot overcome deeper geopolitical divisions. In both cases, the same insight holds: the Asian Games can amplify political claims, but they cannot override the strategic conditions under which those claims are judged. As Murray (56) argues, sport has a “unique power” to bring people, nations, and communities closer together, but that power remains contingent and politically fraught.

The strategic value of sport diplomacy therefore lies in shaping perceptions and testing influence among audiences whose responses are uncertain or constrained. It allows positive images to reach beyond friendly audiences and creates limited opportunities for contact with actors who might otherwise remain distant. These effects do not resolve geopolitical conflict, but they show how sport can open modest, controlled spaces for influence and interaction.

Ultimately, the Asian Games do not remove rivalry; they reveal how conflicts are framed, managed, and politically negotiated in contemporary Asia. Their significance lies not in suspending disputes but in making rivalry visible and actionable in symbolically dense ways. That is why the Asian Games deserve to be taken seriously in political analysis. They show how states in Asia pursue status, seek recognition, and compete over regional order under conditions of deepening uncertainty.

## Data Availability

The original contributions presented in the study are included in the article/Supplementary Material, further inquiries can be directed to the corresponding author/s.
